# Parallel Evolution of Group B *Streptococcus* Hypervirulent Clonal Complex 17 Unveils New Pathoadaptive Mutations

**DOI:** 10.1128/mSystems.00074-17

**Published:** 2017-09-05

**Authors:** Alexandre Almeida, Isabelle Rosinski-Chupin, Céline Plainvert, Pierre-Emmanuel Douarre, Maria J. Borrego, Claire Poyart, Philippe Glaser

**Affiliations:** a Institut Pasteur, Unité Ecologie et Evolution de la Résistance aux Antibiotiques, Paris, France; b CNRS UMR 3525, Paris, France; c Université Pierre et Marie Curie, Paris, France; d Service de Bactériologie, Centre National de Référence des Streptocoques, Groupe Hospitalier Paris Centre Cochin-Hôtel Dieu-Broca, Assistance Publique Hôpitaux de Paris, Paris, France; e DHU “Risques et Grossesse,” Assistance Publique Hôpitaux de Paris, Paris, France; f INSERM, U1016, Paris, France; g CNRS (UMR 8104), Paris, France; h Université Paris Descartes, Sorbonne Paris Cité, Paris, France; i National Institute of Health, Lisbon, Portugal; University of California, Davis

**Keywords:** CovR, ST17, antibiotic resistance, eubacteria, evolution, genomics, group B *Streptococcus*, virulence, GBS vaccine

## Abstract

Group B *Streptococcus* (GBS) is a commensal of the gastrointestinal and genitourinary tracts, while a prevailing cause of neonatal disease worldwide. Of the various clonal complexes (CCs), CC17 is overrepresented in GBS-infected newborns for reasons that are still largely unknown. Here, we report a comprehensive genomic analysis of 626 CC17 isolates collected worldwide, identifying the genetic traits behind their successful adaptation to humans and the underlying differences between carriage and clinical strains. Comparative analysis with 923 GBS genomes belonging to CC1, CC19, and CC23 revealed that the evolution of CC17 is distinct from that of other human-adapted lineages and recurrently targets functions related to nucleotide and amino acid metabolism, cell adhesion, regulation, and immune evasion. We show that the most distinctive features of disease-specific CC17 isolates were frequent mutations in the virulence-associated CovS and Stk1 kinases, underscoring the crucial role of the entire CovRS regulatory pathway in modulating the pathogenicity of GBS. Importantly, parallel and convergent evolution of major components of the bacterial cell envelope, such as the capsule biosynthesis operon, the pilus, and Rib, reflects adaptation to host immune pressures and should be taken into account in the ongoing development of a GBS vaccine. The presence of recurrent targets of evolution not previously implicated in virulence also opens the way for uncovering new functions involved in host colonization and GBS pathogenesis.

**IMPORTANCE** The incidence of group B *Streptococcus* (GBS) neonatal disease continues to be a significant cause of concern worldwide. Strains belonging to clonal complex 17 (CC17) are the most frequently responsible for GBS infections in neonates, especially among late-onset disease cases. Therefore, we undertook the largest genomic study of GBS CC17 strains to date to decipher the genetic bases of their remarkable colonization and infection ability. We show that crucial functions involved in different steps of the colonization or infection process of GBS are distinctly mutated during the adaptation of CC17 to the human host. In particular, our results implicate the CovRS two-component regulator of virulence in the differentiation between carriage- and disease-associated isolates. Not only does this work raise important implications for the ongoing development of a vaccine against GBS but might also drive the discovery of key functions for GBS adaptation and pathogenesis that have been overlooked until now.

**Author Video**: An author video summary of this article is available.

## Abstract (precis)

The incidence of group B *Streptococcus* (GBS) neonatal disease continues to be a significant cause of concern worldwide. Strains belonging to clonal complex 17 (CC17) are the most frequently responsible for GBS infections in neonates, especially among late-onset disease cases. Therefore, we undertook the largest genomic study of GBS CC17 strains to date to decipher the genetic bases of their remarkable colonization and infection ability. We show that crucial functions involved in different steps of the colonization or infection process of GBS are distinctly mutated during the adaptation of CC17 to the human host. In particular, our results implicate the CovRS two-component regulator of virulence in the differentiation between carriage- and disease-associated isolates. Not only does this work raise important implications for the ongoing development of a vaccine against GBS but might also drive the discovery of key functions for GBS adaptation and pathogenesis that have been overlooked until now.

## INTRODUCTION

*Streptococcus agalactiae* (group B *Streptococcus* [GBS]) asymptomatically colonizes the gastrointestinal and urinary tracts of 10 to 30% of the human population (1). However, during the mid-20th century, it emerged as one of the main etiological agents of neonatal disease worldwide. In spite of current prophylactic measures, GBS continues to be a prevalent cause of neonatal morbidity and mortality, especially in late-onset disease (LOD) cases that occur after the first week of life (2). The burden of GBS disease is estimated to be around 0.53 infection per 1,000 live births worldwide, with a higher frequency among low-income countries (2). Since the majority of infections are the result of mother-to-child transmission, there is a significant interest in the development of a maternal vaccine to prevent GBS colonization and subsequent infection of the neonate (3–5).

Among the most promising candidates for vaccination is the capsular polysaccharide, a major virulence factor in GBS (3). Of the 10 capsular types and various clonal complexes (CCs) defined by multilocus sequence typing (MLST), it has been consistently shown that there is a strong association between serotype III strains, CC17 in particular, and neonatal disease (6, 7). For instance, a large epidemiological study in sub-Saharan Africa recently showed that CC17 was most frequently found both in mothers asymptomatically carrying GBS and in infected newborns (8). Additionally, the presence of CC17 is particularly correlated with cases of LOD and meningitis (9). Therefore, this hypervirulent clone has been the focus of genetic and functional studies that have been able to identify some of its unique virulence traits. The colonization and infection ability of GBS relies principally on three mechanisms: (i) the ability to colonize and cross tissue barriers within the host environment, (ii) the ability to evade the host defense mechanisms; and (iii) the expression of virulence factors that cause damage to the host (10). CC17 strains harbor a specific adhesin, termed the hypervirulent GBS adhesin HvgA, that facilitates the crossing of the blood-brain barrier (11). Moreover, unique variants of the serine-rich repeat protein (Srr2) and its SecA2/Y2 secretion system have also been shown to promote the adhesion of CC17 to human epithelial cells (12). Lastly, among the alpha-like family of surface proteins present in GBS, it was observed that CC17 strains carry only the Rib variant (13), which has been reported as an important contributor to the virulence potential of GBS and to elicit protective immunity (14).

The surge in genome sequencing has provided new insights into the population structure and epidemiology of GBS in humans (8, 15–17). Genomic analyses have revealed that the emergence of the main human-associated CCs occurred in the 1950s following the acquisition of the tetracycline resistance determinant *tetM* (15). As tetracycline is no longer used, it remains unclear which adaptive changes allowed the dissemination of the most successful GBS clones detected today. In particular, one important question is how the evolution of hypervirulent CC17 strains might be implicated in their unique ability to colonize and infect the human host.

Here, we combined genomic and evolutionary approaches in the study of 626 GBS strains belonging to CC17 to highlight the specific evolutionary changes selected during adaptation to the human host. We show that disease-specific CC17 isolates frequently acquire mutations that may modulate different stages of GBS pathogenesis, reflecting their distinctive propensity to cause disease in humans.

## RESULTS

### Geographic distribution and clinical association.

To obtain a detailed overview of the genomic diversity of CC17, we compiled all of the publicly available genomes belonging to CC17 as of May 2017 (8, 15, 17–20), together with a new panel of 45 strains, totaling a set of 626 GBS sequences (see Table S1 in the supplemental material). This comprises strains collected between 1955 and 2016 in Africa (*n* = 359), Asia, (*n* = 14), Europe (*n* = 131), North America (*n* = 95), and Australia (*n* = 3), as well as others from unknown origins (*n* = 24). For 94% of the strains (*n* = 586), there was additional information available on whether they came from an asymptomatic carrier or an infected patient. A total of 306 strains were obtained from carriage, and 280 isolates were obtained from infections: 121 (43%) from LOD, 56 (20%) from early-onset disease, 41 (15%) from adults, and 62 (22%) from other or unknown origins. For comparison, we additionally included in our study other publicly available GBS genomes belonging to CC1 (*n* = 368), CC19 (*n* = 241), and CC23 (*n* = 314) (8, 15, 16, 18, 19).

10.1128/mSystems.00074-17.5 TABLE S1  GBS strains analyzed in this work belonging to CC17. Download TABLE S1, XLSX file, 0.1 MB. Copyright © 2017 Almeida et al. 2017 Almeida et al. This content is distributed under the terms of the Creative Commons Attribution 4.0 International license.

### Core genome phylogenetic analyses.

**FIG 1:**
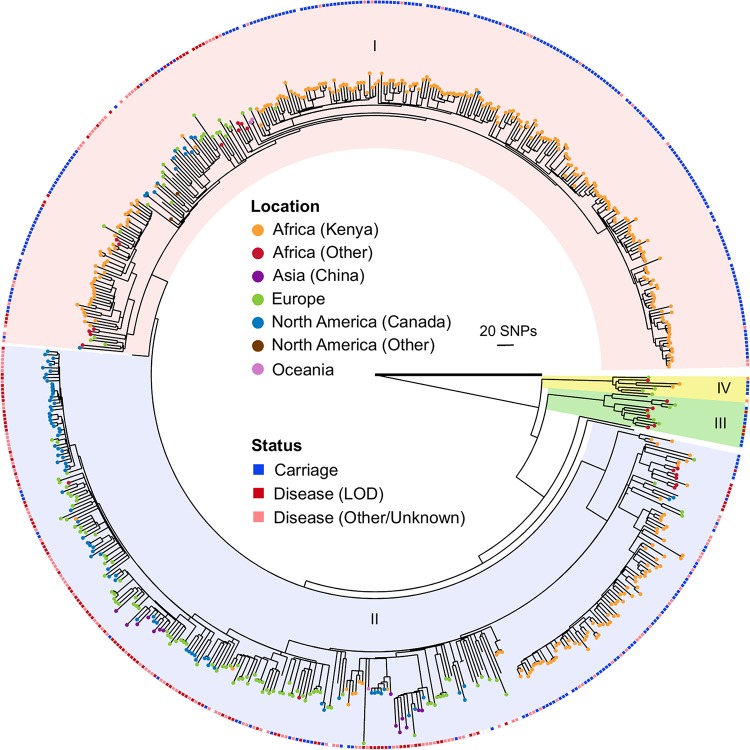
Core genome phylogeny of CC17. Shown is a phylogenetic tree of 626 CC17 genomes built with RAxML (22) and based on the core and recombination-free alignment of 12,584 SNPs along a 1.47-Mb sequence. Outgroup strain B83 is depicted with a thicker branch. Isolates are color coded according to the geographical origin (branch tips) and clinical state (outside circle), as indicated in the central key. The four main distinct clades in the phylogeny are differently colored and labeled with roman numerals.

To assess the structure of the CC17 population, we built a maximum-likelihood (ML) phylogenetic tree of the 626 CC17 genomes (Fig. 1) based on the core and recombination-free alignment of 12,584 single nucleotide polymorphisms (SNPs). We found that one of the novel strains included in this work (B83, isolated in 1970) represents a reliable outgroup of the CC17 population, as it diverged before the main expansion of this clone and the acquisition of the tetracycline resistance determinant *tetM* (15). The phylogenetic tree shows that the CC17 isolates are structured into four main clusters, with most isolates represented within two key lineages (clades I and II). Isolates obtained from infections are found in all four clades, suggesting that disease-associated lineages can arise from distinct genomic backgrounds. We did not detect any additional recombination events with non-CC17 strains, apart from the two previously described regions leading to cluster IV (15, 21) (Fig. 1). We also scanned all of the acquired mutations in each isolate to identify potential recombination events within CC17. Of the 16,572 mutations detected, 502 were found to be homoplasic. By examining how these mutations clustered within the chromosome, we found evidence to suggest that 46 of these homoplasic mutations have arisen through three independent recombination events. We predict that these events, involving regions 25 to 880 kb in length, have occurred between CC17 strains specifically from Kenya (Fig. S1).

10.1128/mSystems.00074-17.1 FIG S1  Homoplasic mutations detected within the CC17 population. Plot of the 502 homoplasic mutations detected in the 626 CC17 strains against their core genome phylogeny. Mutations are arranged according to a hierarchical clustering. Dark green boxes indicate mutation presence, while the three colored overlays depict three putative independent recombination events. Event 1 (light green), 13 mutations between bp 29313 and 909465; event 2 (light red), 20 mutations between bp 98486 and 876971; event 3 (light blue), 13 mutations between bp 260683 and 895533. Download FIG S1, PDF file, 2 MB. Copyright © 2017 Almeida et al. 2017 Almeida et al. This content is distributed under the terms of the Creative Commons Attribution 4.0 International license.

### Intercontinental transmission and worldwide dissemination.

The information available on the geographic origin and isolation date of each strain allowed us to perform a temporal and phylogeographic analysis of the CC17 isolates to examine their transmission in both space and time (Fig. S2). Following the expansion of the four main clones around the 1950s, we estimate that transmission of CC17 strains from Europe to North America seems to have occurred most frequently, with an average of 22 transitions predicted from random simulations based on the phylogeny (Fig. S2B). Also notable were exchanges between Africa and Europe, from North America to Europe, and from Asia (China) to North America (Fig. S2B). Although these conclusions are based on the data available, which have temporal and geographical gaps, they show that CC17 strains have disseminated worldwide through repeated travels across different continents, highlighting their ability to spread easily between human hosts.

10.1128/mSystems.00074-17.2 FIG S2  Intercontinental transmission events and ancestral reconstruction of geographic origins. (A) Plot of the most likely ancestral state (location) predicted for each node of the timed phylogeny of CC17 with the make.simmap tool (58) from the phytools R package (59). (B) the five most frequent intercontinental transmission events inferred with the count.simmap function (58) by counting the transitions between the locations predicted for each node in the phylogeny and the ones at the tips of the tree. Colored circles depict different locations according to the key. Download FIG S2, PDF file, 0.3 MB. Copyright © 2017 Almeida et al. 2017 Almeida et al. This content is distributed under the terms of the Creative Commons Attribution 4.0 International license.

### Parallel and adaptive evolution in the CC17 population.

**FIG 2:**
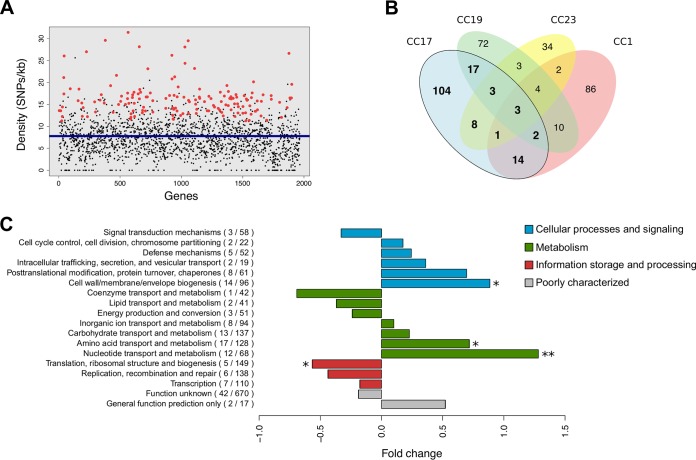
Parallel evolution of coding sequences above neutral expectation. (A) Mutation frequency per gene observed in the CC17 population (black) in relation to the average substitution rate across the COH1 reference genome (blue line). Red dots correspond to the 152 genes with a statistically significant (*P* < 0.05, exact Poisson test) mutational bias compared to a neutral model of evolution (Table S2). (B) Venn diagram depicting the proportion of genes with a significant mutational signature across CC17, CC1, CC19, and CC23. (C) Functional classification of the 152 significant genes in CC17, based on the eggNOG database (24). Fold change corresponds to the proportional difference in the number of genes among those with a mutational bias compared to the COH1 coding sequences (in parentheses). Statistical significance was assessed with a Fisher exact test. *, *P* < 0.05; **, *P* < 0.01.

The frequency and type of genomic mutations fixed in a population provide a record of evolutionary pressures driving adaptation. First, to detect genes with a mutational bias, we compared the expected number of mutations acquired per gene under a neutral model of evolution with that observed in the CC17 population (Fig. 2). From this analysis, we found that a total of 152 genes accumulated significantly more mutations than expected (*P* < 0.05, exact Poisson test; Table S2). When a similar approach was applied to the CC1, CC19, and CC23 GBS populations (Table S3), only three of these significant genes—involved in purine biosynthesis, energy production, and sodium transport—displayed a mutational bias across the four CCs (Fig. 2B). In contrast, 104 (68%) were highly mutated solely among CC17 strains. Globally, pathways involved in cell wall biogenesis and nucleotide or amino acid metabolism were the most affected (*P* < 0.05, Fisher exact test; Fig. 2C), with major virulence-associated factors listed among the genes recurrently mutated (Table S2). CovS, the histidine kinase of the CovR two-component regulator of virulence, acquired 26 independent mutations and was one of the loci with a statistically significant mutational bias after correction for multiple testing. In line with this, the serine/threonine protein kinase Stk1, which also regulates CovR activity (22), was highly affected by accumulating a total of 26 mutations. Among the most frequent targets of parallel and convergent evolution were several components of the bacterial cell envelope, such as the *dltD* gene, which is part of the *dlt* operon responsible for d-alanylation of the lipoteichoic acids within the cell wall; two class C sortases of pilus island 1 (PI-1), the fibrinogen-binding protein FbsA, and three genes from the *cps* operon (*cpsD*, *cpsE*, and *cpsG*) involved in the biosynthesis of the capsule. Notably, the gene for serine-rich repeat protein Srr2, unique to CC17 strains, was among the most significantly mutated genes, with 55 independent substitutions.

10.1128/mSystems.00074-17.6 TABLE S2  Genes with a statistically significant mutational bias detected in CC17. Download TABLE S2, XLSX file, 0.04 MB. Copyright © 2017 Almeida et al. 2017 Almeida et al. This content is distributed under the terms of the Creative Commons Attribution 4.0 International license.

10.1128/mSystems.00074-17.7 TABLE S3  Genes with a statistically significant mutational bias detected in CC1, CC19, and CC23. Download TABLE S3, XLSX file, 0.1 MB. Copyright © 2017 Almeida et al. 2017 Almeida et al. This content is distributed under the terms of the Creative Commons Attribution 4.0 International license.

Next, we looked for evidence of adaptive evolution by searching for genes that accumulated more amino acid-modifying mutations than expected, with the canonical test of natural selection (ratio of nonsynonymous to synonymous evolutionary changes [*dN*/*dS*]). Genes related to the bacterial cell envelope coding for PI-1 ancillary protein 1, the streptococcal histidine triad protein (GBSCOH1_RS08985), and a protein similar to zoocin A (GBSCOH1_RS00315) presented a statistically significant signal of positive selection (*P* < 0.05) with the acquisition of 20, 19, and 13 amino acid changes, respectively, relative to no synonymous substitutions. Besides being strongly regulated by CovRS, PI-1 has been suggested to play a role in GBS resistance to phagocytic killing specifically by macrophages (23). However, PI-1 has also been independently lost by particular CC17 lineages (17, 20), so its contribution to GBS adaptation is still unclear and is likely multifaceted. Another target of adaptation, the streptococcal histidine triad protein, contributes to GBS pathogenesis by facilitating the evasion of complement-mediated host immune responses (24). Finally, zoocin A is a peptidoglycan endopeptidase that was previously described in *Streptococcus equi* subsp. *zooepidemicus* (25), but its role in GBS remains unknown.

We similarly searched for mutations enriched within noncoding sequences and identified 85 regions with a higher-than-expected number of mutations (*P* < 0.05, exact Poisson test; Table S4). Among these, there was a significant bias of mutated sequences upstream of CovR-repressed targets (26) (*P* < 0.01, Fisher exact test). Those coding for the hypervirulent GBS adhesin HvgA and CovR itself were among the 10 targets most frequently affected upstream. Of note were also 14 mutations upstream the *fbsA* and *srr2* genes, together with 21 upstream of *fbsB* (Table S4), which further evidences the *in vivo* selection of modifications potentially affecting the function of these surface proteins. One recently described noncoding RNA (Srn073) (27), located between GBSCOH1_RS08135 and GBSCOH1_RS08140 (IR66, Table S4), was also detected as a frequent target of evolution (*n* = 19 mutations).

10.1128/mSystems.00074-17.8 TABLE S4  Noncoding regions with a statistically significant mutational bias detected in CC17. Download TABLE S4, XLSX file, 0.03 MB. Copyright © 2017 Almeida et al. 2017 Almeida et al. This content is distributed under the terms of the Creative Commons Attribution 4.0 International license.

### Convergent pathoadaptive mutations among disease-associated lineages.

To find a potential link between specific polymorphisms of the CC17 strains and their pathogenicity, we devised a strategy to extract the mutations exclusively acquired by carriage or disease isolates (see Materials and Methods). A total of 5,818 or 6,771 mutations were uniquely identified among isolates collected from carriers or infected patients, respectively. Of the putative pathoadaptive mutations, we identified 10 homoplasic substitutions affecting genes directly associated with virulence (Table S5). This signal of convergent evolution was observed in the zoocin A protein, in PI-1, and in loci related to cell adhesion (*fbsA* and *rib*), capsule biosynthesis and regulation (*neuD*, *neuC*, *cpsE*, *cpsL*, and *cpsD*), and virulence regulation (*covS*; Table S5).

10.1128/mSystems.00074-17.9 TABLE S5  Convergent mutations associated with disease. Download TABLE S5, XLSX file, 0.04 MB. Copyright © 2017 Almeida et al. 2017 Almeida et al. This content is distributed under the terms of the Creative Commons Attribution 4.0 International license.

**FIG 3:**
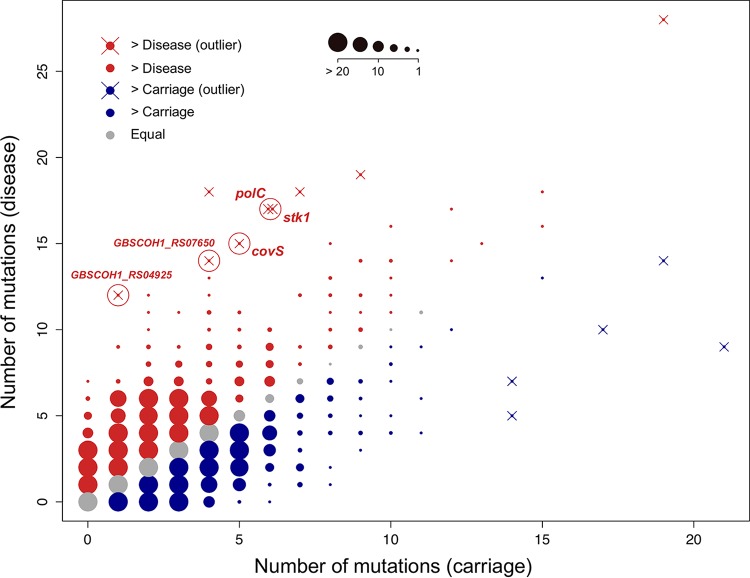
Mutation frequencies per gene in carriage- and disease-associated isolates. Shown is a plot of a linear model assessing the correlation between the mutations acquired per gene for each clinical status. Red, gray, and blue dots depict genes that were more, equally, or less mutated in strains associated with disease or carriage according to the key at the top. The size of each data point is proportional to the number of genes found, as indicated in the key at the top. Outlier genes were detected with a Bonferroni-adjusted outlier test, and only those with a *P* value of <0.05 are represented by the symbol ×. Those highlighted with a red circle and whose names are shown were significantly associated with disease independently of population structuring.

**FIG 4:**
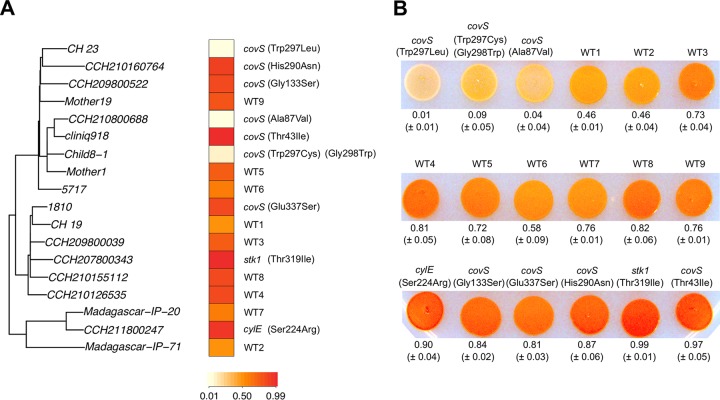
Pigmentation of strains with *covRS*-related mutations. (A) Level of pigment production plotted against the phylogeny of 18 different CC17 strains. Strains WT1 to WT9 correspond to isolates with no mutations in genes assumed to affect the activity of CylE (*covR*, *covS*, *abx1*, *stk1*, and *cylE*). For the remaining strains, mutations potentially affecting *covRS* or *cylE* are indicated next to the heat map. Strains WT1, WT2, and WT4 to WT9 were obtained from carriers, while all others were collected from infections. (B) Plate image of the strains tested, with average values and standard deviations of the pigment levels obtained from four independent experiments depicted below the spots. Ratios ranging from 0 to 1 were the result of normalization against the sample with the highest intensity in each test, calculated with ImageJ (https://imagej.nih.gov/ij).

After correlating the numbers of mutations acquired per gene by carriage and disease isolates, we identified 14 outlier genes whose mutation frequencies differed significantly (*P* < 0.05, Bonferroni-adjusted outlier test) between the carriage and infection scenarios (Table 1; Fig. 3). Five of them were significantly skewed toward disease independently of the population structuring of clades I and II (Fig. 1). Among the genes with the most disease-associated mutations were those coding for the kinases Stk1 and CovS (Table 1). The CovRS system is known to regulate the hemolytic activity of GBS and the production of an orange pigment through the *cyl* operon (28, 29). By looking at the degree of pigmentation in a panel of 18 strains—9 mutants and 9 with no mutations in genes potentially affecting the expression or activity of *cyl* (*covR*, *covS*, *abx1*, *stk1*, and *cylE*)—we observed extreme levels of pigmentation (≤0.09 and ≥0.97; Fig. 4) across different strains that acquired nonsynonymous substitutions in *covS* or *stk1*. Moreover, two of the least pigmented strains acquired convergent SNPs affecting the same amino acid within CovS (Trp297; Fig. 4). Hence, we speculate that these mutations might have an impact on the phenotype of CC17 strains through the modulation of CovR activity.

**TABLE 1:** Genes most distinctly mutated between carriage- and disease-associated isolates

Locus	Product	Disease^a^	Carriage^a^	*P* value^b^
GBSCOH1_RS00285	Phosphoribosylformylglycinamidine synthase	19 (11/8)	9 (6/3)	6.6900 × 10^−4^
GBSCOH1_RS01850^c^	Serine/threonine protein kinase Stk1	17 (13/4)	6 (3/3)	4.7100 × 10^−4^
GBSCOH1_RS04925^c^	23S rRNA methyltransferase	12 (7/5)	1 (1/0)	0.0115
GBSCOH1_RS04975	ABC transporter permease	18 (15/3)	4 (1/3)	7.1600 × 10^−7^
GBSCOH1_RS05095	Cell division protein FtsK	28 (23/5)	19 (12/7)	4.5700 × 10^−6^
GBSCOH1_RS06600	Cell wall anchor Srr2	18 (10/8)	7 (6/1)	2.3500 × 10^−4^
GBSCOH1_RS07650^c^	Amidase	14 (9/5)	4 (2/2)	0.0161
GBSCOH1_RS07705^c^	Two-component sensor histidine kinase CovS	15 (11/4)	5 (5/0)	0.0088
GBSCOH1_RS08845^c^	DNA polymerase III subunit alpha PolC	17 (10/7)	6 (4/2)	4.7100 × 10^−4^
GBSCOH1_RS04480	Type II CRISPR RNA-guided endonuclease Cas9	14 (8/6)	19 (13/6)	6.4900 × 10^−5^
GBSCOH1_RS05130	Carbamoyl phosphate synthase large subunit	10 (8/2)	17 (15/2)	4.5100 × 10^−5^
GBSCOH1_RS06095	Peptidase C5	9 (7/2)	21 (13/8)	2.8400 × 10^−12^
GBSCOH1_RS08245	X-prolyl-dipeptidyl aminopeptidase	5 (3/2)	14 (11/3)	1.2200 × 10^−4^
GBSCOH1_RS08960^c^	Hypothetical protein	7 (5/2)	14 (12/2)	2.3800 × 10^−3^

a Number of mutations exclusively acquired by strains associated with each clinical state. Values in parentheses correspond to the numbers of nonsynonymous and synonymous substitutions detected, respectively.

b Bonferroni-adjusted *P* values were obtained with the outlier test.

c Gene with a mutation frequency significantly biased toward either carriage or disease, independent of association with clade I or II (Fig. 1).

### Phase variation in the surface protein Rib.

**FIG 5:**
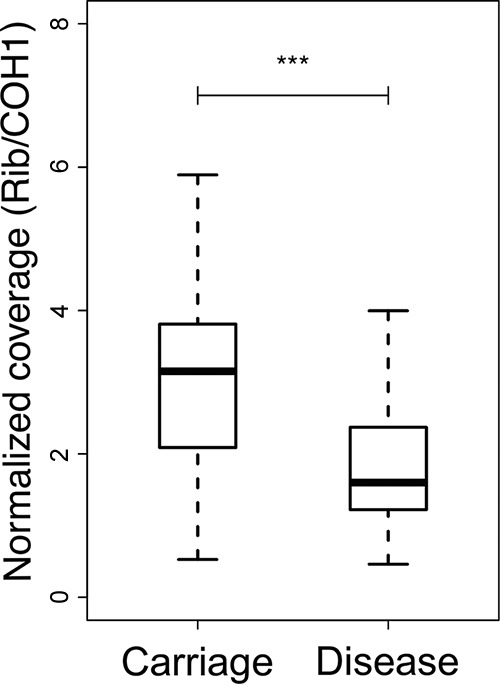
Phase variation of *rib* between carriage and disease. Shown is the normalized coverage of the gene coding for the alpha-like surface protein Rib in carriage- and disease-specific strains, as also depicted in Fig. S3. Statistical significance values were calculated with a two-tailed *t* test. ***, *P* < 0.001.

Diversity within a population not only stems from nucleotide-level mutations (SNPs or indels) but might also result from the presence of gene copy number variation (CNV), which ultimately affects fitness and evolutionary outcomes. After normalizing the coverage distribution with that of reference strain COH1 isolated from disease, we identified 115 genes that displayed at least double the sequence coverage in at least one strain and 56 after excluding those with unknown functions or related to mobile genetic elements (Fig. S3). Although the variation observed across the phylogeny for some of these genes could have been influenced by mapping noise, that of the gene coding for the Rib protein was markedly increased in various isolates. We found that the coverage estimated for *rib* was a direct correlation of the number of tandem repeating motifs following its unique N-terminal part. There was no association between the coverage of *rib* and the phylogeny, which is indicative of a flexible adaptability typical of phase variation. On the other hand, by comparing carriage-related isolates with disease-associated strains, we observed a significant reduction (*P* < 0.001, two-tailed *t* test) in the number of repeat units in Rib among strains from infections (Fig. 5).

10.1128/mSystems.00074-17.3 FIG S3  Coverage distribution of genes with functional relevance. Heat map depicting the coverage variation of 56 genes across the CC17 phylogeny colored according to clinical status, as represented in Fig. 1. Values correspond to the sequencing coverage normalized with that obtained with the self-mapped reference genome of COH1. The *rib* gene is indicated by an arrow. Download FIG S3, PDF file, 0.3 MB. Copyright © 2017 Almeida et al. 2017 Almeida et al. This content is distributed under the terms of the Creative Commons Attribution 4.0 International license.

### Acquisition and spread of antibiotic resistance genes.

**FIG 6:**
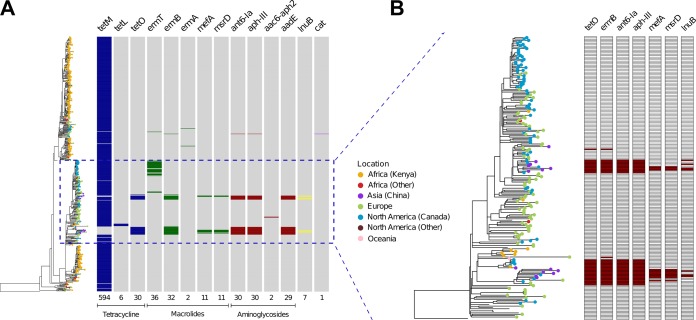
Genetic characterization of antibiotic resistance. (A) Antibiotic resistance determinants present in the ResFinder database (47) that were detected in both the sequencing reads and the assembled genomes of the CC17 strains, plotted according to their core genome phylogeny. The number of isolates with each antibiotic resistance gene is depicted below each column. Gene absence is represented in gray, while the remaining colors illustrate the different classes of antibiotics. (B) Distribution of the antibiotic resistance genes present within a genomic island that was first detected among CC17 strains from China (20). The phylogenetic tree corresponds to a particular clade containing all 14 CC17 strains that have been sequenced in China (20). For each gene, dark red boxes indicate presence and white indicates absence.

As antibiotic resistance is known to be involved in the successful dissemination of pandemic clones, we probed the presence of antibiotic resistance determinants in the CC17 genomes (Fig. 6). The *tetM* gene conferring tetracycline resistance is widespread across the CC17 population (present in 95% of the isolates), which emphasizes both its crucial role in the dissemination of these strains and its stable integration. Besides tetracycline resistance, macrolide and aminoglycoside resistance traits were the ones most abundantly found, but to a much smaller degree (Fig. 6A). These resistance genes are also phylogenetically constrained, having been acquired only by specific clades within the CC17 population. A recent study found that CC17 strains in China frequently carry a genomic island harboring a multidrug resistance gene cluster (20). Our genomic comparison of these samples showed that they branch into three clades with other isolates collected from Africa, Europe, and Canada that also carry this element (Fig. 6B). Despite sampling limitations in the studies that were included in this work, the distribution of these isolates along the phylogeny of CC17 illustrates independent acquisition events and a recent flux of multidrug-resistant GBS strains most frequently from China to Canada (Fig. 6B; Fig. S2).

### Analyzing the unique genetic repertoire of CC17.

Complementing the SNP-based evolutionary analysis of the CC17 population, we compared the gene contents of GBS genomes from various CCs to identify regions that could be associated with the hypervirulent properties of CC17. By analyzing the pangenome of 32 complete GBS genomes belonging to 17 different sequence types (STs), we identified a total of 1,553 core genes (present in ≥95% of the strains) and 2,589 accessory genes. Of those, 79 were acquired specifically by CC17 genomes and up to one other ST, while a group of 23 genes are present in most CCs but absent from CC17 in particular (Table S6; Fig. S4). A remaining set of 27 genes is distributed among a few different clones alongside CC17, reflecting a more ambiguous history of genetic gain and loss. Looking at the functions potentially gained by horizontal exchange, we confirmed the acquisition of *hvgA*, *srr2*, and *rib* by CC17, together with several genes coding for uncharacterized proteins (Table S6). Of those, we identified putative cell surface proteins, kinases, proteases, and lipoproteins. This suggests that beyond the virulence traits already characterized in CC17, other genes were also selected in this lineage and might contribute to its pathogenicity.

10.1128/mSystems.00074-17.4 FIG S4  Distribution of genes differentially present between CC17 and other CCs. Plot of 129 genes that are either significantly present in or absent from the CC17 lineage against the phylogeny of 32 complete GBS genomes. These include all of the finished genomes belonging to CC17 that were available as of May 2017. The association between gene content and CC17 was inferred with Scoary (https://github.com/AdmiralenOla/Scoary), and the phylogeny was built from the core genome alignment of the assembled genomes with Parsnp (54). Download FIG S4, PDF file, 0.1 MB. Copyright © 2017 Almeida et al. 2017 Almeida et al. This content is distributed under the terms of the Creative Commons Attribution 4.0 International license.

10.1128/mSystems.00074-17.10 TABLE S6  Accessory genes associated with CC17. Download TABLE S6, XLSX file, 0.03 MB. Copyright © 2017 Almeida et al. 2017 Almeida et al. This content is distributed under the terms of the Creative Commons Attribution 4.0 International license.

### Investigating the persistence of GBS in neonates.

**FIG 7:**
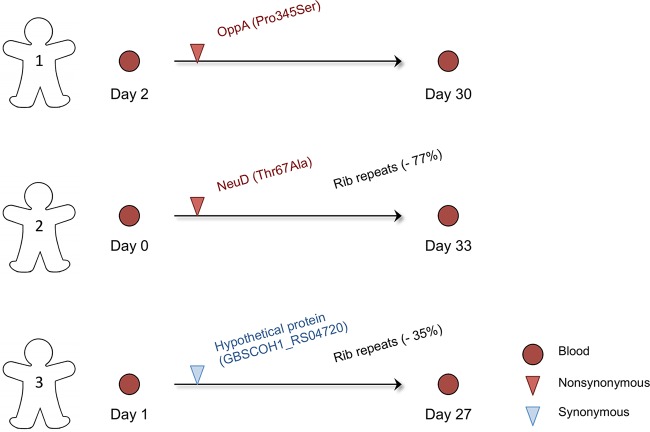
Persistence and short-term evolution of CC17 in neonates. Shown are mutations differentiating three pairs of isolates collected 1 month apart from different blood cultures of infected newborns. The number of days since birth is indicated below each strain. The name of the gene affected by the mutation and its effect on the protein sequence are presented as shown in the key at the lower right. Percentages alongside Rib represent changes in normalized sequencing coverage.

To gain insights into a potential genetic basis for the persistence of CC17 in the newborn, we characterized the adaptive changes inferred from longitudinal samples collected 1 month apart from three newborns with GBS-positive blood cultures (Fig. 7). Isolates obtained at both time points were genotypically related, with only one SNP having been acquired in the sample collected 1 month later (Fig. 7). This means that the relapsed infection of the bloodstream was the result of GBS strains deriving from the ones originally infecting each newborn. Strikingly, a missense mutation detected in the *neuD* gene involved in capsular sialylation (Thr67Ala; Table S5) is a convergent mutation independently acquired by another isolate that was collected from the cerebrospinal fluid of a neonatal meningitis patient (K38783; Table S1). Also notable was that for two of the pairs of strains, the number of repeats in the gene coding for the Rib surface protein was lower in the strain collected at the second time point (Fig. 7), which is further evidence of evolutionary pressures in the newborn selecting for the reduction of Rib repeats during infection.

## DISCUSSION

Strains of hypervirulent CC17 are the most prevalent cause of GBS neonatal disease worldwide, but the reasons for their evolutionary success remain poorly understood. In this work, we leveraged the data obtained from all of the CC17 genomes available to date to track the parallel and convergent evolution of multiple clinical isolates and to characterize the distinguishing traits between carriage- and disease-specific strains. We identified a consistent bias in the frequency and type of genomic mutations affecting genes with pivotal roles during the infection or colonization process of GBS, namely, for cell adhesion, host immune evasion, and virulence regulation (Table 2). The presence of multiple targets of adaptation also underscores the multifactorial pathogenesis of GBS infections, in that disease outcome is determined by the intricate relationship between host- and pathogen-specific properties.

**TABLE 2:** Recurrent targets of evolution related to the bacterial cell envelope and implicated in GBS virulence

Locus	Product	Function	Significance^a^
GBSCOH1_RS00315	Zoocin A	Unknown	CMC, MBC, PS
GBSCOH1_RS01850	Serine/threonine protein kinase Stk1	Virulence regulation	CMC, DA, MBC
GBSCOH1_RS02505	Alpha-like surface protein Rib	Cell adhesion	CMC, CMU, DA, MBU
GBSCOH1_RS03235	PI-1 transcriptional regulator	Immune evasion	CMC
GBSCOH1_RS03240	PI-1 backbone protein	Immune evasion	CMC, CMU
GBSCOH1_RS03250	PI-1 class C sortase	Immune evasion	CMC, MBC
GBSCOH1_RS03255	PI-1 class C sortase	Immune evasion	MBC
GBSCOH1_RS03260	PI-1 ancillary protein 1	Immune evasion	PS
GBSCOH1_RS05180	Fibronectin-binding protein FbsA	Cell adhesion	CMC, CMU, MBC, MBU
GBSCOH1_RS05705	NeuD protein	Immune evasion	CMC
GBSCOH1_RS05710	NeuC protein	Immune evasion	CMC
GBSCOH1_RS05720	Capsular polysaccharide transporter CpsL	Immune evasion	CMC
GBSCOH1_RS05745	β-1,4-Galactosyltransferase CpsG	Immune evasion	CMC, MBC
GBSCOH1_RS05755	Galactosyltransferase CpsE	Immune evasion	CMC, MBC
GBSCOH1_RS05760	Tyrosine protein kinase CpsD	Immune evasion/regulation	CMC, MBC
GBSCOH1_RS06095	Peptidase C5	Immune evasion	CMC, MBC
GBSCOH1_RS06600	Cell wall anchor Srr2	Cell adhesion	CMC, CMU, MBC, MBU
GBSCOH1_RS07705	Sensor histidine kinase CovS	Virulence regulation	CMC, DA, MBC
GBSCOH1_RS07710	DNA-binding response regulator CovR	Virulence regulation	CMC, MBU
GBSCOH1_RS08490	Extramembranal protein DltD	Resistance to AMPs^b^	CMC, MBC
GBSCOH1_RS08985	Streptococcal histidine triad protein	Cell adhesion/immune evasion	PS
GBSCOH1_RS09645	Hypervirulent GBS adhesin HvgA	Cell adhesion	CMC, CMU, MBU

a Evidence suggesting that the gene is a significant target of evolution, based on our evolutionary analyses. CMC, convergent nonsynonymous mutation within the coding sequence; CMU, convergent mutation upstream; DA, disease association; MBC, mutational bias within the coding sequence; MBU, mutational bias upstream; PS, positive selection.

b AMPs, antimicrobial peptides.

Most of the major human CCs were selected in the 1950s after the acquisition of the tetracycline resistance determinant *tetM* (15). Moreover, their evolution was shown to have been mediated primarily by the exchange of large chromosomal regions (15, 30). In the case of CC17, the only two recombination events we have detected with a non-CC17 background occurred before the acquisition of *tetM* and led to a serotype IV lineage that has until now never been associated with LOD, a hallmark of CC17 strains. In contrast, we inferred that three recombination events occurred between geographically related serotype III CC17 strains (Fig. S1). This observation supports the hypothesis that since the expansion of its two major clones, CC17 isolates corresponds to a genetically isolated lineage (15), occupying a specific niche in the digestive tract with little interaction with other GBS clones.

Genetic variation naturally occurring within a population can be selected differently between distinct genomic backgrounds due to inherent features of each species or clone. Of the genes we detected under parallel evolution, the majority have a significant mutational signature exclusively in the CC17 population compared to other human-associated CCs (Fig. 2B). The genomic repertoire of CC17 may carry a unique potential to colonize and infect the human host, so mutations that subsequently arise are differentially selected in other backgrounds, even if under similar evolutionary pressures. Nevertheless, PI-1 and the *cps* operon, two important surface components identified here as evolutionary hot spots in the CC17 population, are also found to be under diversifying selection in the CC1 lineage, as previously described (16). However, PI-1 in particular is also absent from certain CC17 lineages (17, 20). Many components of the cell envelope are frequent targets for adaptation in CC17 (Table 2). Importantly, there is a possible association between a convergent nonsynonymous mutation in *neuD* with the persistence of GBS and the etiology of meningitis, both intrinsically related to the incidence of LOD (Fig. 7; Table S5). It is also noteworthy that the expression of *neuD* has been described as being higher specifically in strains obtained from cerebrospinal fluid (31). Also present on the bacterial surface is the protein Rib, whose highly immunogenic properties are inversely correlated with the number of its repeated domains (32). Therefore, we speculate that strains with a smaller Rib—detected preferentially in infected newborns—are able to more readily escape the antibodies maternally transmitted and be strongly selected in the course of disease, as was previously observed in a mouse model of infection (33). Similarly, we had formerly reported that a reduction in the expression of Rib could present a selective advantage in neonatal disease following maternal transmission (18).

The two-component CovRS system is a major regulator of most virulence-associated genes in GBS (26). We identified frequent nucleotide substitutions in the CovS sensor histidine kinase predominantly among disease-associated strains (Tables 1 and 2). Strikingly, a mutational bias was also detected in the serine/threonine kinase Stk1, which regulates CovR, and in the upstream regions of *covRS* and nine of its targets, including *hvgA*. Stk1 mediates CovRS activity through phosphorylation of the CovR response regulator (22, 29), which in turn reduces its ability to bind to target promoter regions of various virulence-associated genes (e.g., *hvgA*), derepressing their transcription. We predict that the whole CovRS regulatory pathway represents a key target for the adaptation of CC17 strains, as slight genomic changes can have dramatic phenotypic repercussions (34, 35). Similarly, in group A *Streptococcus*, it has been shown that mutations in regulatory networks, such as *covRS*, may underlie the phenotypic heterogeneity observed between strains from infection and asymptomatic carriage (36).

Although we stress the importance of genomic variation affecting traits for which there is extensive knowledge, our work also paves the way for the further characterization of genes not previously implicated in virulence. One such example is the gene coding for a protein similar to zoocin A that has only been studied in *S. zooepidemicus* and was among the three genes with a statistically significant signature of nonsynonymous substitutions in the CC17 population (Table 2). Pangenome analyses of fully sequenced GBS genomes also revealed that the CC17 lineage acquired a number of genes with as-yet-uncharacterized functions that may further contribute to its hypervirulence (Table S6; Fig. S3).

In conclusion, we present a thorough genomic analysis of the *in vivo* evolution of CC17 and identify genetic traits converging toward important phenotypes involved in the adaptation and pathogenicity of GBS in humans. These results provide a greater understanding of the adaptive evolutionary changes that have underpinned the global dissemination of this lineage following the emergence of the main human-associated clones in the 1950s (15). The development of a GBS vaccine has been identified as a high priority for the World Health Organization (5) and could ostensibly reduce the incidence of CC17 as a cause of neonatal disease. However, we found that the most promising candidates for a GBS vaccine (4), such as the capsule, the pilus, and Rib, are frequent targets of evolution. Therefore, this high level of adaptability will need to be carefully considered in the development of preventive strategies against GBS colonization.

## MATERIALS AND METHODS

### Bacterial strains.

A total of 626 GBS genomes belonging to CC17 were analyzed in this work (Table S1). Among those, 581 genomes were obtained by surveying public databases for all of the available GBS sequences belonging to CC17. In brief, an *in silico* MLST analysis of all of the GBS genomes accessible as of May 2017 was performed with either SRST2 (37) on the raw sequencing reads or a BLAST-based python script on the assemblies. For the purpose of this work, strains with no more than one allelic difference from the MLST profile of ST17 were considered members of CC17. A total of 561 genomes were selected and retrieved from the published studies of Almeida et al. (18) (*n* = 25), Campisi et al. (20) (*n* = 14), Da Cunha et al. (15) (*n* = 79), Rosini et al. (19) (*n* = 18), Seale et al. (8) (*n* = 333), and Teatero et al. (17) (*n* = 92), together with 20 additional genomes deposited in the NCBI database (Table S1). To complement our analysis of the CC17 clone, a similar approach was used to extract 923 publicly available GBS genomes belonging to CC1, CC19, and CC23 (8, 15, 16, 18, 19). A set of 45 CC17 strains provided by the Centre National de Référence des Streptocoques in France, the Collection de l’Institut Pasteur (CIP), and the pediatric hospital of Luanda (38) were also added and sequenced in this study (Table S1).

### Whole-genome sequencing, assembly, and pangenome analysis.

Chromosomal DNA extraction of the novel set of 45 CC17 isolates was performed with the DNeasy blood and tissue kit (Qiagen). Libraries were prepared by the Nextera XT protocol, and genomes were sequenced with the Illumina HiSeq 2500 platform by using paired-end read runs of ~150 bp. Reads were filtered for quality and then assembled with either Velvet (39) or SPAdes (40). Low-quality assemblies, identified by their total length, high number of contigs, or lack of one of the housekeeping genes, were subsequently discarded. Strain BM110 (Table S1) was additionally selected for single-molecule real-time sequencing (PacBio RS II system). PacBio subreads were assembled with both Canu (41) and the RS_HGAP_Assembly.3 protocol from the SMRT analysis toolkit v2.3, while consensus accuracy was further polished with Quiver (42) as previously described (43). Genome assemblies were annotated with Prokka (44) by using a custom database comprising reference sequences (RefSeq) of GBS and other streptococci. Pangenome analysis of 32 available complete genomes of GBS belonging to various CCs were carried out with Roary (45). Genes with a specific association with CC17 genomes were identified by the Scoary script (https://github.com/AdmiralenOla/Scoary). Detection of antibiotic resistance genes was performed with SRST2 (37) on the sequencing reads and with the large-scale BLAST score ratio (LS-BSR) pipeline (46) on the genome assemblies by using the ResFinder database (47). Each antibiotic resistance gene was considered present if detected in both the raw sequencing data and the assembled genome.

### Genome mapping, variant calling, and phylogenetic analyses.

We used Burrows-Wheeler aligner (BWA) (48) to map the sequencing reads of each CC17 genome against the complete reference sequence of COH1. To analyze parallel evolution in other CCs, we used strain SS1 (NZ_CP010867) as the reference for the CC1 genomes, strain H002 (NZ_CP011329) for the CC19 strains, and NEM316 (NC_004368) for all of the CC23 samples. For those that did not have any raw data available, sequencing reads were simulated from the assembled genome with ART (49). Gene CNV of the CC17 genomes was deduced with the R package CNOGpro (50), based on the BWA mapping of each strain by using raw sequencing data, normalized with that of simulated reads from the reference genome of COH1. Variant calling was performed with Genome Analysis ToolKit v3.4.0 (51) in accordance with the published recommendations (52, 53). Briefly, SNPs were filtered on the basis of the following criteria: alternate allele frequency, >90%; read depth (DP), >10; quality by depth (QD), >2.0; Fisher strand bias (FS), <60.0; mapping quality (MQ), >40.0; mapping quality rank sum test (MQRankSum), greater than −12.5; read position rank sum test (ReadPosRankSum), greater than −8.0.

Phylogeny of all CC17 strains was inferred from the polymorphic positions detected in the variant calling workflows, while the phylogenetic tree comprising the 32 complete GBS genomes was based on the core genome alignment obtained with Parsnp (54). Recombinant sites and accessory genes—missing from more than 1% of the isolates—were removed following identification with Gubbins (55) and the filter_BSR_variome.py script from the LS-BSR pipeline (46), respectively. ML phylogenies were built with RAxML (56) by using a general time-reversible substitution model with a gamma-distributed rate across sites combined with an ascertainment bias.

To investigate the temporal evolution of CC17, the Bayesian phylogenetic software BEAST v2.3.1 (57) was used. The evolutionary rate of this population was calibrated by using the corresponding sampling date of each strain (Table S1) as previously described (15). To characterize the phylogeographic distribution of the CC17 strains and infer possible events of intercontinental transmission, we then used the make.simmap tool (58) from the phytools R package (59). Discrete ancestral traits, matching one of the geographical locations of the samples, were predicted for each node of the CC17 phylogeny by modeling 1,000 simulations. The resulting numbers of intercontinental transitions between nodes and from node to tip were calculated with the count.simmap function (58).

### Variant annotation, parallel evolution, and mutation classification.

To predict the impact of the SNPs detected within coding sequences, snpEff (60) was used to classify each point mutation as either nonsynonymous or synonymous. To detect genomic regions with a mutational bias, accessory and recombined regions were removed solely from the affected strains. If removed from >50% of the target population, they were excluded from all subsequent analysis. In principle, without recombination and assuming a constant mutation rate across the genome, the number of substitutions per gene under neutral evolution can be modeled as a Poisson distribution. Therefore, a signal of parallel evolution was inferred from a statistically significant increase in the substitution rate over that expected under a null hypothesis of neutral evolution, as previously described (61). Multiple testing correction was performed by the Benjamini-Hochberg procedure with a false-discovery rate of 10%. Homoplasic events were detected by searching for mutations that occurred in the same position at least twice in lineages with a minimum of a five-strain gap between them, based on the phylogeny. Genes with a mutational bias in the CC17 population were classified into functional categories by using the eggNOG database v4.5 (62). We then used a Fisher exact test to assess the statistical significance of the functions affected in relation to their overall proportion in the COH1 reference strain. For *dN*/*dS* calculations, the observed spectrum of nonsynonymous (N) and synonymous (S) mutations per gene was normalized by the expected N/S ratio obtained through simulation of all possible nucleotide substitutions in each reference genome. Values of >1 are indicative of positive selection, and statistical significance was assessed with the binomial test.

Variants associated with the available metadata (Table S1) were extracted with VCFtools (63). Mutations were classified as carriage or disease related on the basis of whether they were exclusively present in CC17 isolates collected either from infected individuals or from asymptomatic carriers. This involved removing mutations that arose in ancestral lineages common to both carriage and disease strains. Subsequently, for each locus of the genome of COH1, the total number of mutations classified into each clinical state was calculated. To investigate the genes most differentially mutated, a linear model of correlation was built between carriage and disease mutation frequencies, and the outlier genes were detected with a Bonferroni-adjusted outlier test. Given the greater proportion of carriage-associated strains in clade I (Fig. 1), we took into account the effect of population structuring in our analysis by performing a similar assessment of genes with a mutational bias toward clade I or II (Table 1).

### GBS pigmentation.

To assess the degree of pigmentation of each GBS strain, bacterial cells were cultured overnight in TH broth at 37°C in standing cultures. Subsequently, 7 µl of each strain was spotted onto Granada agar plates (BioMérieux) and grown overnight at 37°C in a CO_2_ environment. Pictures were taken with the plates against a black background, and the contrast was adjusted for easier discrimination of weak and strong pigment producers. The level of pigmentation was measured by the color intensity of each spot and quantified within a circle of the same area size with ImageJ (https://imagej.nih.gov/ij). Values were then normalized against the sample with the highest intensity in each test to generate a ratio of 0 to 1. Experiments were performed in quadruplicate, and the resulting average values and standard deviations are presented in Fig. 4.

### Data availability.

Sequencing reads from the 45 newly sequenced strains and the complete genome assembly of strain BM110 have been deposited in the EMBL nucleotide sequence database (http://www.ebi.ac.uk/ena) under study accession number PRJEB18603.
