# Supplementary figures and images for: Parallel Evolution of Group B Streptococcus Hypervirulent Clonal Complex 17 Unveils New Pathoadaptive Mutations
Source: mSystems. 2017 Sep 5;2(5):e00074-17. doi: 10.1128/mSystems.00074-17 (PMC5585690; doi:10.1128/mSystems.00074-17)

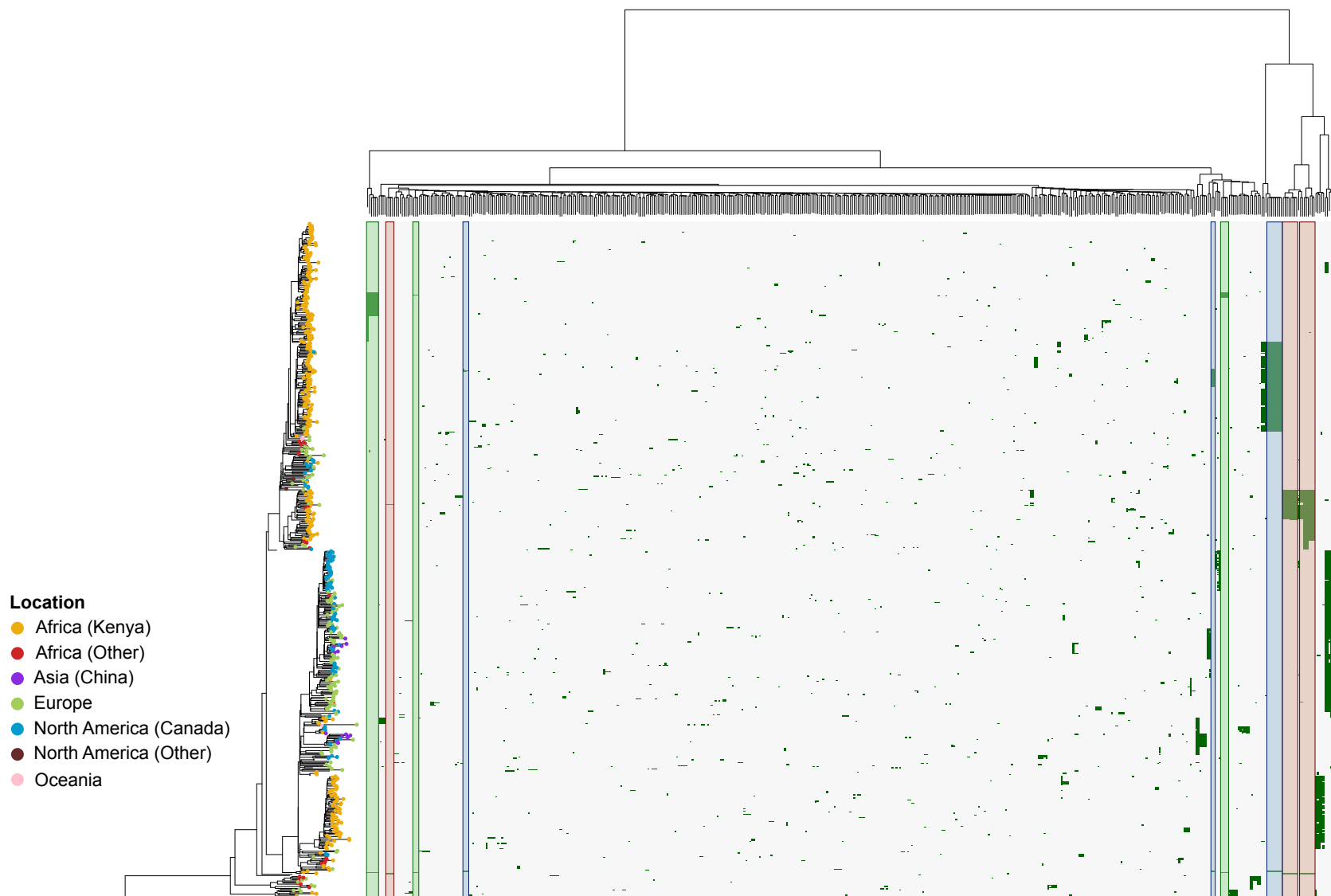

Fig. S1

Supplement: FIG S1 [file sys005172132sf1.pdf]

**A**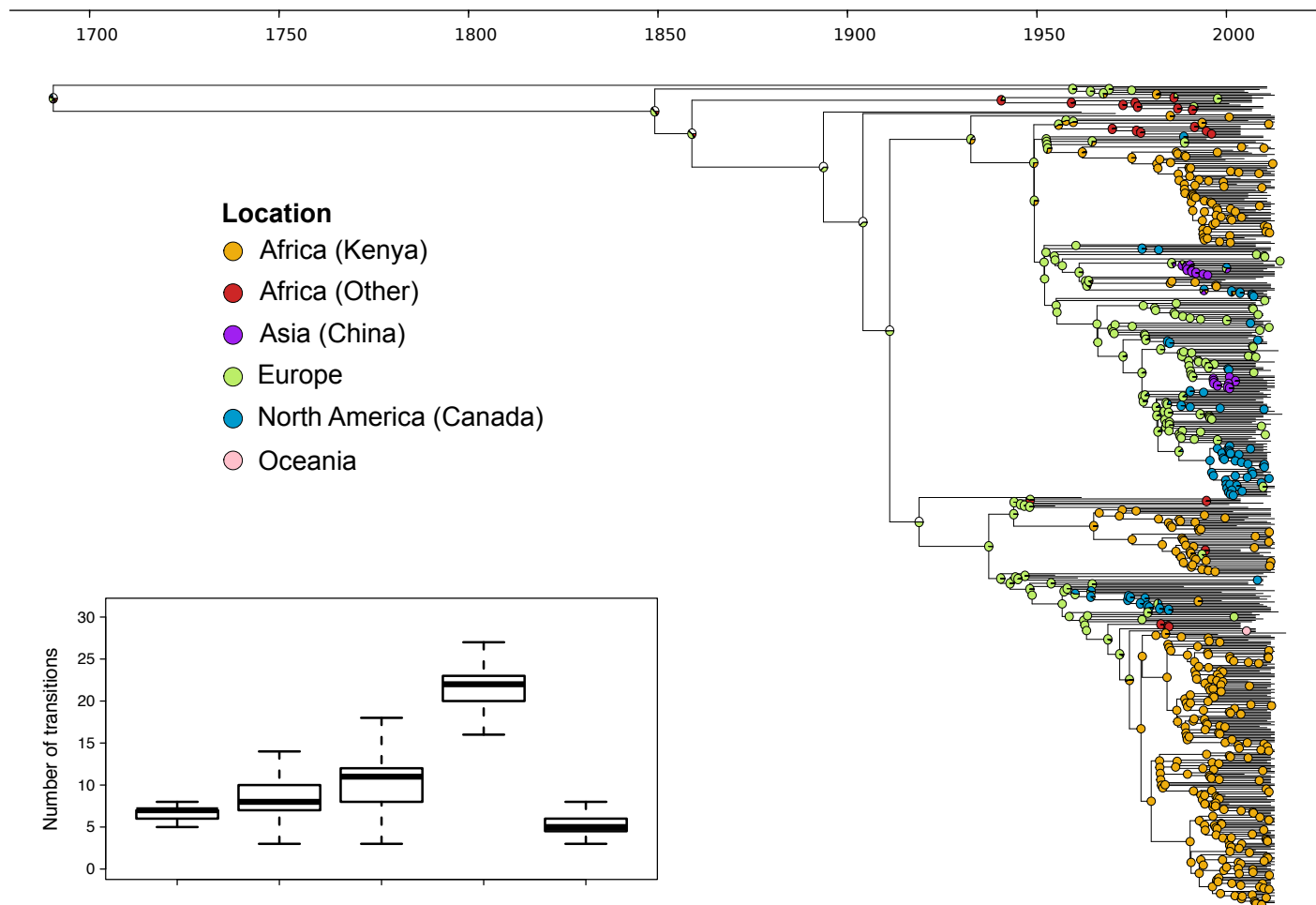**B**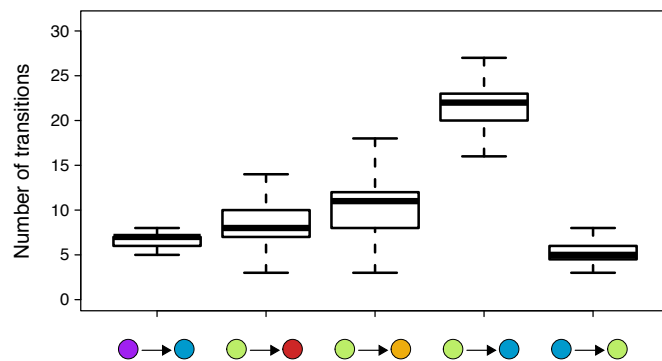

Fig. S2

Supplement: FIG S2 [file sys005172132sf2.pdf]

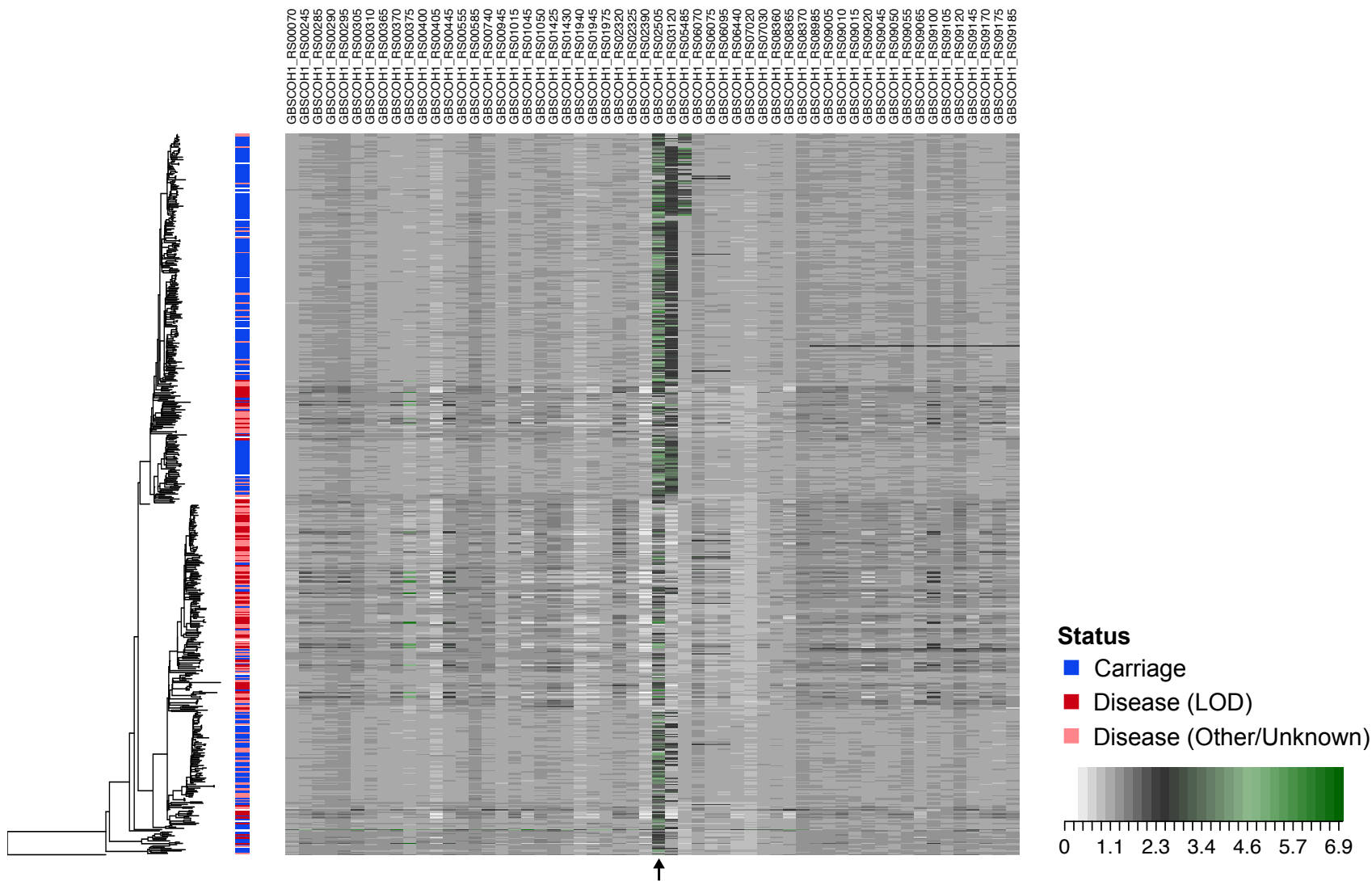

Fig. S3

Supplement: FIG S3 [file sys005172132sf3.pdf]

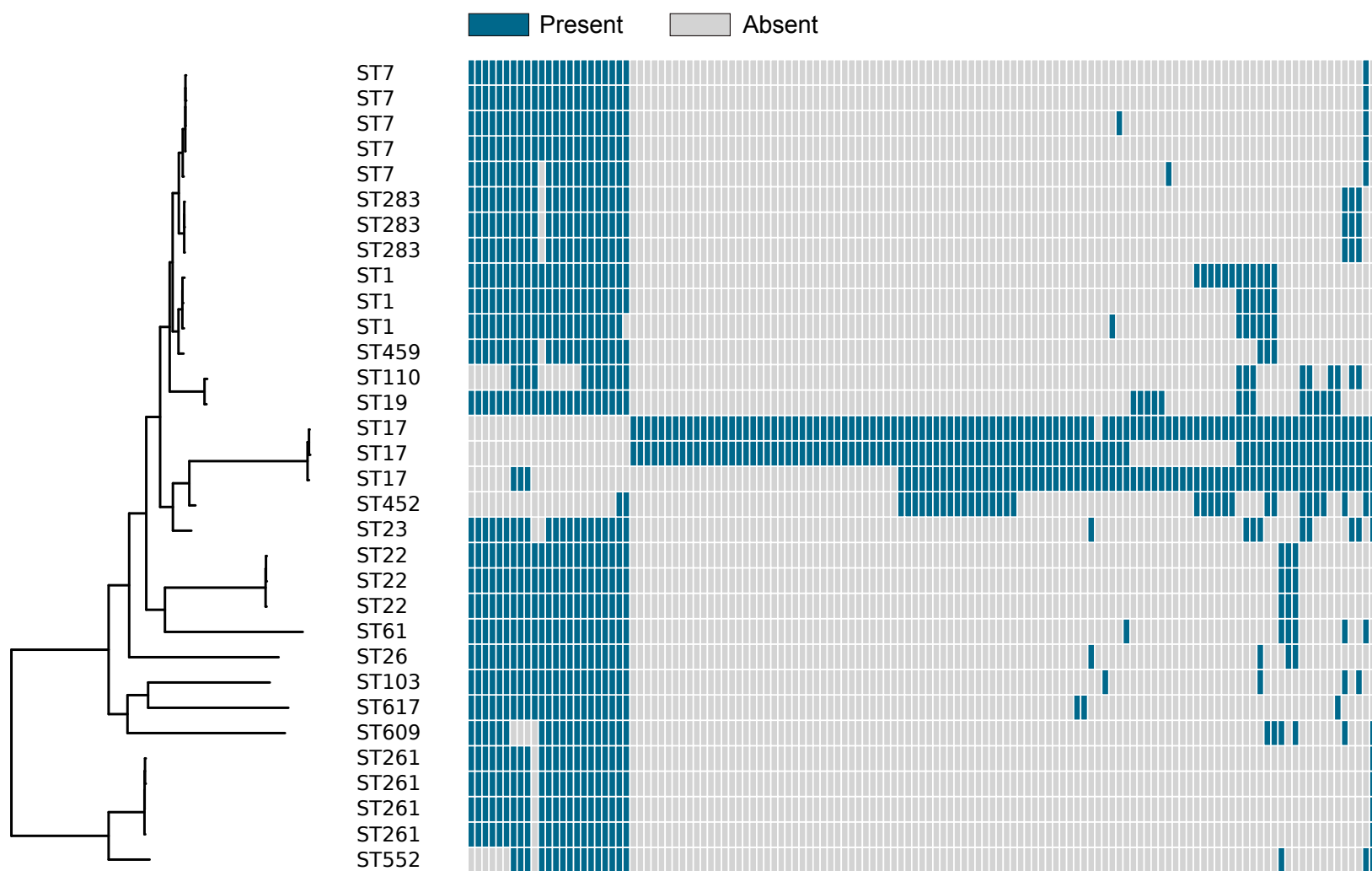

Fig. S4

Supplement: FIG S4 [file sys005172132sf4.pdf]
